# Local Genomic Epidemiology of *Acinetobacter baumannii* Circulating in Hospital and Non-hospital Environments in Kano, Northwest Nigeria

**DOI:** 10.1007/s00284-025-04304-z

**Published:** 2025-06-08

**Authors:** I. Yusuf, H. B. Idris, E. Skiebe, G. Wilharm

**Affiliations:** 1 Department of Microbiology, Faculty of Life Sciences, Bayero University Kano, Kano, Nigeria; 2 Project Group P2, Robert Koch Institute, Burgstr. 37, 38855 Wernigerode, Germany; 3 Microbiology & AMR Research Unit, Kano Independent Research Center Trust, Kano, Nigeria

## Abstract

*Acinetobacter baumannii* is a pathogenic bacterium of public health significance, capable of rapidly spreading within and between environments. The local epidemiology and transmission pattern of *A. baumannii* strains circulating in hospitals and non-hospital environments is rarely studied, and hence this is investigated in Kano, Nigeria. A cross-sectional study design was used to collect 172 samples from clinical, hospital, and non-hospital samples. *Acinetobacter baumannii* isolates were identified and confirmed using microbiological and molecular techniques. Variants of *bla*_OXA-51_ were determined through amplicon sequencing, while whole genome sequencing was performed on 22 isolates to determine their allelic variants/sequence types (ST), resistance/virulence genes, insertion sequences, plasmids, single-nucleotide polymorphism (SNPs) and investigate phylogenetic relationships between the isolates. Twenty-seven *A. baumannii* were isolated from door handle and toilet floors of student hostels (14), soil and sullage samples (3 each), bed, chair, and drawer of hospital environments (9), and 1 from the urine of a patient. All these isolates belong to only 2 variants of *bla*_OXA51_-like genes, 16 (48.8%) are *bla*_OXA-66_ and 11 (33.3%) are *bla*_OXA-180_. About 70% of the isolates were susceptible to many antibiotics, and 8 resistance genes encoding aminoglycoside, tetracycline, and sulphonamide resistance were acquired by only strains harbouring *bla*_OXA-66_, and between 37 and 39 virulence genes were harboured by all the variants. Intrinsic *bla*_ADC-25_ encoding resistance to β-lactams was found in all *A. baumannii* strains. The 2 variants had Pasteur scheme MLST allelic profiles ST2 and 267, which are not commonly reported in Nigeria. Few isolates from hospital and non-hospital sources form a cluster with SNPs number distances within the two clusters in the range of 85–100, suggesting a close relationship. The 2 variants circulate in both environments, suggesting transmission in both directions. Detection of ST267 (*bla*_OXA-180_ variant) in a clinical sample indicates an environment-to-human transmission.

**Supplementary Information:**

The online version contains supplementary material available at 10.1007/s00284-025-04304-z.

## Introduction

*Acinetobacter baumannii* are Gram-negative, obligate aerobic, oxidase-negative, flagella-lacking bacteria that are globally known to cause life-threatening infections such as sepsis, meningitis, blood, urinary tract, skin, soft tissue, and bone infections.

Many hospital strains have been reported to possess the capacity to resist multiple antibiotics and quickly acquire antimicrobial resistance genes (ARGs) from other bacteria and the surrounding environment [1, 2]. Both carbapenem-resistant and susceptible *A. baumannii* have been reported to occupy diverse range of environments, which include hospital surfaces [3], aquatic ecosystems [4], municipal wastewater treatment plants [5], soil [6, 7], and frequently touched surfaces in shared environments [8]. The bacteria can successfully establish themselves in the mentioned environments when they express one or more virulence factors that facilitate their excellent ability to survive in harsh environments, resist desiccation, and form biofilms, the properties that facilitate their epidemic spread within and outside hospital settings. While their transmission between one person and another in hospitals is often through contaminated medical devices, surfaces, utensils, and hands of caregivers [3], in non-hospital settings, transmission is through direct contact, especially through sharing of contaminated surfaces [8]. Transmission in non-hospital environments (especially frequently touched and shared environments) can be facilitated when poor basic infrastructures such as pipe-borne water, solid waste disposal systems, and drainages to convey wastewater generated from residential and schools are available, a phenomenon that can play a role as important as clinical settings for the selection of antibiotic-resistant *A. baumannii* in the environment [9, 10]. In addition, the strains that acquire new genes might harbor chromosome and plasmid combinations that optimize their fitness and transmissibility.

It is also important to note that Africa, including Nigeria, remains significantly underrepresented in global genomic studies of *A. baumannii* [11]. As highlighted in Castillo-Ramírez’s commentary on Müller’s [11] global view on carbapenem-resistant *A. baumannii,* many regions in Africa were not included in the large-scale surveillance efforts due to limited partnerships, lack of sampling infrastructure, or insufficient capacity to collect both susceptible and resistant isolates [12]. While such limitations are understandable, they result in major gaps in the global picture of *A. baumannii* transmission and resistance, which reinforces the need for more genomic investigations from underrepresented regions like Nigeria to better understand the local and global dynamics of this pathogen.

While only a few studies have reported molecular epidemiology of clinical transmission of *A. baumannii* in a hospital in southwestern Nigeria [13, 14], there is presently no information on the local epidemiology, genomic features, and transmission pathways of *A. baumannii* strains colonizing hospital and non-hospital environments in Nigeria. To clarify the role of environment-to-person, and non-hospital-to-hospital transmission, and vice versa, we investigated the local epidemiology and genetic relatedness of 22 *A. baumannii* isolates recovered from different sources using whole genome sequencing data. We evaluated their antibiotic resistance phenotypes, genes encoding carbapenem, aminoglycoside, tetracycline, beta-lactam and sulphonamide resistance, insertion sequence (IS) elements, virulence-associated genes and plasmids maintained by the *A. baumannii* isolates.

## Materials and Methods

### Sample Collections and Microbiological Analysis

In the study, samples from the hospital environment, non-hospital environment, and clinical samples were collected over a period of 6 months. Samples of the hospital environment were collected by swabbing surfaces of chairs, beds, and drawers used by patients admitted into the medical and surgical wards of Aminu Kano Teaching Hospital (AKTH) and Murtala Muhammad Specialist Hospital (MMSH) using COPAN Amies agar gel medium transport swabs (COPAN 108C and 110C). For non-hospital environments, samples of soil were collected from a semi-pristine ecological and botanical garden in Bayero University Kano, Nigeria, while samples of grey water (sullage) freely discharged into the environment from shared student hostels were collected into sterile containers. In addition, swabs of room door handles and toilet floors shared by students were also collected from five blocks of hostels. Clinical urine samples directly from patient or from urine bags were taken and transported to the laboratory.

Swabs were inoculated directly into MacConkey agar (MCA). Five grams of each soil sample was transferred into a sterile 50 ml bottles, and 5 ml of mineral salt medium supplemented with 0.2% acetate was added and incubated for 6 h (and up to 24 h) in a rotary shaker (enrichment) before inoculation on to the MCA. The composition of the mineral salt medium per liter of water is 10 g KH_2_PO_4_, 5gNa_2_HPO_4_,2 g (NH4)_2_SO_4_, 0.2 g MgSO_4_.7H_2_O, 0.001 g CaCl_2_ (2H_2_O), 0.001 g FeSO_4_.7H_2_O. Typical *A. baumannii* colonies were assessed from overnight incubated plates by lactose fermentation and morphological appearances. Gram reaction and biochemical reactions were used to distinguish *A. baumannii* grown on MAC from other Gram-negative bacteria. The typical colonies were further inoculated onto CHROMagar Acinetobacter (CHROMagar, France) for further confirmation. From the confirmed colonies, genomic DNA was extracted using boiling method, and PCR technique was used to amplify the *bla*_OXA51-like_ genes to confirm *A. baumannii* from other *Acinetobacter* spp. as previously reported [15, 16].

### Antimicrobial Susceptibility Testing

Susceptibility of confirmed *A. baumannii* isolates to different antibiotics which include ampicillin (AMP), ceftazidime (CAZ), cefepime (FEP), piperacillin-tazobactam (PTZ), tetracycline (TET), tigecycline (TGE), imipenem (IMP), gentamicin (C), kanamycin (K), colistin (CT), rifampicin (RD), ciprofloxacin (CIP) and chloramphenicol (CN) was tested using the disc diffusion according to CLSI guidelines with result interpretation according to CLSI 2020.

### Genotypic Characterization of *A. baumannii* Variants

The full-length sequences of *bla*_OXA-51*like*_ genes were then determined by amplifying the region with primers OXA-69A (5′-CTAATAATTGATCTACTCAAG-3′) and OXA-69B (5′-CCAGTGGATGGATGGATAGATTATC-3′) [17] followed by Sanger sequencing using BigDye terminator cycle sequencing. Raw reads were de novo assembled and the variant type was determined in NCBI data base using blastx, a tool that searches protein databases using a translated nucleotide query.

### Whole Genome Sequence Analysis

The whole genomic DNA of the bacteria was extracted with the Genomic DNA purification kit (Zymos research, US) and quantified using the Qubit™ dsDNA BR Assay Kit (Invitrogen, Waltham, MA, United States). The values from Qubit device was then adjusted by appropriate dilution with dilution buffer to obtain sample concentrations in the range of 10–15 ng/µl. DNA library was prepared using the Nextera XT DNA Library Prep Kit according to the manufacturer’s standard protocols (Illumina Inc., USA). Paired-end sequencing of the generated libraries was carried out on the Illumina MiSeq sequencer (Illumina, USA). Quality checks of raw sequencing data were performed using FastQ and multiQC [18, 19]. Sequences for each isolate were assembled de novo using Velvet v1.20.10 [20]. The completeness and contamination of the assembled genomes were assessed using CheckM2 v1.0.2 and QUAST v5.2.0 [21, 22]. Preliminary analysis to estimate the taxonomic composition of the bacteria samples was carried out using the kraken tool against the MiniKraken database. To confirm the taxonomic classification of the isolates, average nucleotide identity (ANI) values were determined between the genomes of each strain and the genomes of their respective type strains using fastANI v1.33 [23, 24]. Genome annotation was performed using the NCBI Prokaryotic Genome Annotation Pipeline (PGAP) [24].

To determine and fully describe the allelic variants of all 7 housekeeping gene fragments in all the isolates, all the assembled genomes were typed using the MLST web-server and BacWGSTdb (BacWGSTdb (bacdb.cn)) against the Oxford and Pasteur schemes [25]. In addition, intrinsic and acquired antibiotic resistance genes, virulence genes, as well as single-nucleotide polymorphism (SNPs) between the isolates were determined using BacWGSTdb. In addition to BacWGSTdb, ResFinder (https://cge.cbs.dtu.dk/services/ResFinder/) was also used to determine the resistance genes in the assembly. The threshold for reporting a match between a gene in the ResFinder database and the input sequence was set at a 90% identity with a minimum length of 60%. The cgMLST *A. baumannii* scheme was defined with BacWGSTdb data using *A. baumannii* AYE as the reference genome. Insertion sequences, and plasmids were derived using IS Finder [26], and Plasmid Finder [27], respectively. Plasmid reconstruction, typing, and mobilization prediction was performed using MOB-suite v3.1.9 with the default settings [28]. For IS elements, IS*Aba1*, IS*Aba2*, IS*Aba3*, IS*Aba125*, IS*Aba825*, and IS*Aba11* are more targeted because they have been shown to be associated with enhanced resistance to penicillins, cephalosporins, carbapenems, and colistin in clinical *A. baumannii* isolates. Core genome phylogeny was constructed from concatenated sequences of core gene alignments generated using Panaroo version 1.3.4 [29]. The resulting phylogenetic tree was annotated and visualized using iTOL (Interactive Tree of Life, itol.embl.de) [30]. A total of 68 genomes of *A. baumannii* were included in the analysis, which represent isolates from diverse non-clinical and clinical sources across different geographic regions of the globe.

## Results

### Phenotypic and Genotypic Characterization of *A. baumannii*

*Acinetobacter baumannii* was isolated from all the sampling sites at varying rates. Out of the total 172 samples obtained from the 3 sources between Dec 2020 and June 2021, *Acinetobacter spp.* were recovered from 34 samples. Morphological analysis of *Acinetobacter spp* from swab samples directly inoculated on MAC and later on CHROMagar Acinetobacter (CMA) media revealed typical lactose non -fermenting (on MCA) or bright red (on CMA), smooth, convex colonies. For soil samples, typical colonies of *A. baumannii* were clearly observed when the samples were enriched in mineral salt media + 0.2% sodium acetate for 6–24 h. Characterization results showed *A. baumannii* to be Gram-negative, oxidase-negative, non-flagellated, and cocco-bacilli bacteria.

**Fig. 1:**
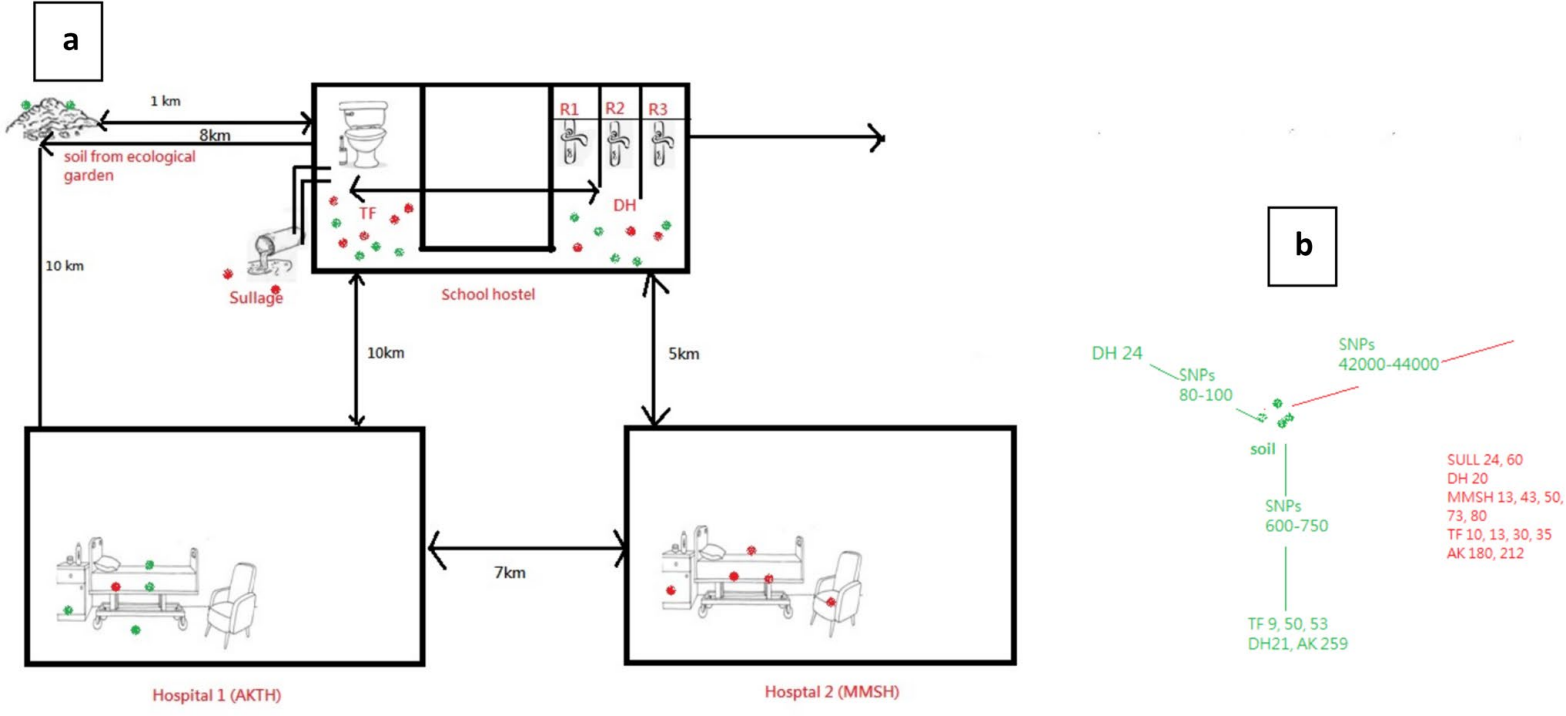
The distribution of the 2 variants of *bla*_OXA51_ like genes (i.e. *bla*_OXA-66_ and *bla*_OXA-180_) across all the sites sampled is shown in this figure. **a** Soil samples from semi pristine environment harboured only the *bla*_OXA-180_ (green). In school hostel which is about 1–2 km from the soil sampling sites, the 2 variants are present in the toilet floor and door handles shared by many students. In hospital 1, isolates harbouring*bla*_OXA-180_ are obtained from bed, drawer surfaces, while in hospital 2, which is about 7 km from 1, isolates contain *bla*_OXA-66_ (red) in all the surfaces sampled. **b** The *A. baumannii* isolates recovered from soil exhibited close genetic relatedness, with 80–100 SNPs compared to those isolated from door handle (DH_24) in a hostel

From 50 samples of door handles (DH) and toilet floors (TF), 5 (10%), and 13 (26%) *A. baumannii* were isolated, respectively. In addition, 3 *A. baumannii* were isolated from 10 samples of sullage (Sull), and from 12 samples of soil collected from the ecological and botanical gardens (Fig. 1). A total of 9 isolates were recovered from 30 hospital environmental samples as follows: 5 from the bed, 1 from a chair, and 3 from the drawers. From clinical samples, 1 *A. baumannii* was isolated from urine (Table 1). Amplification of *bla*_OXA-51 like_ genes was positive in 27 isolates. On sequencing the *bla*_OXA-51_like gene, 2 variants of *bla*_OXA-51_ like genes were identified (*bla*_OXA-66_ and *bla*_OXA-180_). 15 *A. baumannii* isolated from 4 hospital beds, 2 drawers, 5 toilet floors, 2 sullage, 1 door handle, and 1 chair encodes protein variant *bla*_OXA-66_, while *bla*_OXA-180_ was obtained from 12 isolates as follows, soil sample (*n* = 1), toilet floors (*n* = 5), door handle (*n* = 3), hospital drawer (*n* = 1), hospital bed (*n* = 1), and urine sample (*n* = 1) (Table 2).

**Table 1:** Isolation of *Acinetobacter spp* from different hospital and non-hospital sources

Sample site	Specific site	No. of samples collected	No. positive	%
Student hostel	Door handle	50	5	10
	Toilet floor	50	13	26
	Sullage	10	3	30
Soil samples	Ecological garden	6	2	33.3
	Botanical garden	6	1	16.7
Clinical samples	FMW Urine	10	1	10
	Wound	10	0	0
Hospital Environment	Bed swab	10	5	50
	Chair swab	10	1	10
	Drawer swab	10	3	30
Total		172	34	19.8

**Table 2:** Sampling sites and *A. baumannii* variants

Sample ID	Source of sample	Ward/location	Date of collection	Specific sampling site	OXA-51 variant
MMSH_13	Murtala Muhd Specialist Hospital	Female medical ward	12.12.20	Bed	66
MMSH_43	Murtala Muhd Specialist Hospital	Male medical ward	11.01.21	Bed	66
AK_180	Aminu Kano Teaching Hospital	Surgical ward	18.01.21	Drawer	180
BB_1	Ecological garden	Soil	12.07.21	Ecological garden	180
AK_311	Aminu Kano Teaching Hospital	Male medical ward	01.03.21	Bed	180
AK_212	Aminu Kano Teaching Hospital	Female surgical Ward	08.03.21	Drawer	66
AK_259	Aminu Kano Teaching Hospital	Male surgical ward	22.03.21	Urine	180
TF_10	Bayero University student hostel	Male hostel	11.01.21	Toilet floor	66
DH_20	Bayero University student hostel	Female hostel	05.04.21	Door handle	66
TF_13	Bayero University student hostel	Female hostel	13.12.20	Toilet floor	66
TF_31	Bayero University student hostel	Female hostel	17.12.20	Toilet floor	180
MMSH_80	Murtala Muhd Specialist Hospital	Female surgical ward	09.08.21	Bed	66
MMSH_73	Murtala Muhd Specialist Hospital	Female surgical ward	03.05.21	Drawer	66
TF_9	Bayero University student hostel	Male hostel	17.05.21	Toilet floor	180
DH_21	Bayero University student hostel	Male hostel	17.05.21	Door handle	180
TF_50	Bayero University student hostel	Male hostel	07.06.21	Toilet floor	180
TF_53	Bayero University student hostel	Male hostel	14.06.21	Toilet floor	180
TF_54	Bayero University student hostel	Male hostel	28.06.21	Toilet floor	180
DH_24	Bayero University student hostel	Male hostel	05.07.21	Door handle	180
Sull_60	Bayero University student hostel	Male hostel	19.07.21	Sullage	66
Sull_24	Bayero University student hostel	Male hostel	26.07.21	Sullage	66
TF_30	Bayero University student hostel	Male hostel	26.07.21	Toilet floor	66
TF_27	Bayero University student hostel	Male hostel	12.07.21	Toilet floor	66
MMSH_50	Murtala Muhd Specialist Hospital	Male medical ward	09.08.21	Bed	66
MMSH_69	Murtala Muhd Specialist Hospital	Male medical ward	21.08.21	Chair	66
DH_34	Bayero University student hostel	Male hostel	09.08.21	Door handle	180
TF_45	Bayero University student hostel	Male hostel	12.07.21	Toilet floor	66

In contrast, the soil isolate showed a much higher distance, with 600–750 SNPs relative to isolates from another door handle isolate (DH21) toilet floors (TF9, 50, 53) and AKTH_259 which was isolated from hospital environment. Additionally, a much greater genetic distance was observed between the soil isolates and *bla*_OXA-66_-carrying isolates from sullage collected from close vicinity, DH_20, TF_10,13,30,35, AKTH_180, 212, and MMSH_13,43,50,73,80, which suggest environmental persistence and the potential movement of strains across hospital and non-hospital settings.

### Phenotypic Antimicrobial Resistance

*Acinetobacter baumannii* isolated from all the sources showed phenotypically varying resistance to different antibiotics. Apart from natural resistance to ampicillin, resistance to chloramphenicol, kanamycin and tetracycline reached 66, 77.7, 74%, respectively. Only a single *A. baumannii* hospital bed isolate showed resistance (zone of inhibition, 10 mm) to imipenem. Four (33.3%) out of the 12 *bla*_OXA-180_ variants are susceptible to kanamycin. Nearly all the isolates were susceptible to colistin, tigecycline, and rifampicin, and intermediate susceptibile to gentamicin. Four and one *bla*_OXA-66_ and *bla*_OXA-180_ variants, respectively, were resistant to ceftazidime, and 6 environmental samples (both hospital and non-hospital environment) are resistant to cefepime (Table 3).

**Table 3:** Antibiotic susceptibility pattern of *A. baumannii* isolates against different antibiotics

S/N	Sample ID	Site/sample	OXA51 variant	C	CN	K	CT	IMP	TE	TGE	AMP	RD	CAZ	CIP	PTB	FEP
				Zone of inhibitions (mm) according to CLSI standards												
1	AKTH_212	Drawer	66	**0 ± 0**	**12 ± 0.0**	0 ± 0	15 ± 3.1	26 ± 2.2	**0 ± 0**	16 ± 2.0	**0 ± 0**	16 ± 0.5	20 ± 2.6	31 ± 0.5	**0 ± 0**	14 ± 2.6
2	DH_20	Door handle	66	**0 ± 0**	**11 ± 1.3**	0 ± 0	14 ± 0.4	26 ± 3.2	**0 ± 0**	16 ± 1.4	**0 ± 0**	17 ± 0.5	21 ± 0.5	16 ± 0.0	**8 ± 0**	**9 ± 0.2**
3	MMSH_13	Bed	66	**0 ± 0**	**10 ± 3.2**	0 ± 0	15 ± 2.2	26 ± 1.3	**0 ± 0**	15 ± 0.2	**0 ± 0**	18 ± 2.1	**10 ± 0.0**	19 ± 0.0	**0 ± 0**	**11 ± 2.0**
4	MMSH_43	Bed	66	**0 ± 0**	**11 ± 0.0**	**0 ± 0**	14 ± 1.3	26 ± 2.4	**0 ± 0**	14 ± 1.2	**0 ± 0**	17 ± 5.7	**9 ± 0.0**	**0 ± 0**	**0 ± 0**	**0 ± 0**
5	MMSH_50	Bed	66	**0 ± 0**	**11 ± 0.8**	**0 ± 0**	15 ± 2.8	24 ± 2.2	**0 ± 0**	14 ± 0.0	**0 ± 0**	16 ± 2.5	**9 ± 0.0**	**0 ± 0**	**0 ± 0**	**0 ± 0**
6	MMSH_69	Chair	66	**12 ± 1.2**	15 ± 0.1	**0 ± 0**	19 ± 3.4	28 ± 1.7	**0 ± 0**	20 ± 0.0	**0 ± 0**	20 ± 2.1	**11 ± 1.5**	**0 ± 0**	**0 ± 0**	23 ± 2.4
7	MMSH_ 73	Drawer	66	**0 ± 0**	**13 ± 1.4**	**0 ± 0**	14 ± 2.2	25 ± 0.8	**0 ± 0**	18 ± 1.8	**0 ± 0**	20 ± 0.4	**10 ± 3.1**	**0 ± 0**	**0 ± 0**	**0 ± 0**
8	MMSH_80	Bed	66	18 ± 0.0	14 ± 1.4	**0 ± 0**	20 ± 1.0	29 ± 1.1	**0 ± 0**	20 ± 0.4	**0 ± 0**	20 ± 1.6	24 ± 0.0	**0 ± 0**	26 ± 2.2	22 ± 1.2
9	Sull_24	Sullage	66	**0 ± 0**	**12 ± 0.0**	**0 ± 0**	14 ± 0	23 ± 2.0	**0 ± 0**	12 ± 2.1	**0 ± 0**	14 ± 3.2	24 ± 0.0	**0 ± 0**	**0 ± 0**	**0 ± 0**
10	Sull_60	Sullage	66	**8 ± 0**	**13 ± 0.1**	**0 ± 0**	14 ± 2.1	25 ± 3.5	**0 ± 0**	15 ± 3.5	**0 ± 0**	14 ± 2.5	22 ± 0.0	**14 ± 0.0**	**0 ± 0**	**10 ± 0.0**
11	TF_10	Toilet floor	66	**0 ± 0**	**11 ± 2.7**	**0 ± 0**	16 ± 0.9	24 ± 1,8	23 ± 0.8	16 ± 2.0	**0 ± 0**	15 ± 0.5	29 ± 1.2	**13 ± 0**	**0 ± 0**	26 ± 0.0
12	TF_13	Toilet floor	66	**0 ± 0**	**11 ± 0.3**	**0 ± 0**	15 ± 1.2	25 ± 0.4	20 ± 1.0	17 ± 2.4	**0 ± 0**	16 ± 2.5	29 ± 2.0	18 ± 0.6	**0 ± 0**	27 ± 1.0
13	TF_27	Toilet floor	66	**0 ± 0**	**12 ± 2.1**	**0 ± 0**	16 ± 2.0	24 ± 0.8	**0 ± 0**	19 ± 3.2	**0 ± 0**	15 ± 2.2	28 ± 1.0	22 ± 0.5	**0 ± 0**	28 ± 0.0.0
14	TF_30	Toilet floor	66	**0 ± 0**	**10 ± 2.5**	**0 ± 0**	15 ± 0.0	26 ± 1.6	**0 ± 0.1**	15 ± 1.2	**0 ± 0**	16 ± 1.2	27 ± 0	23 ± 1.5	**0 ± 0**	27 ± 0.5
15	TF_45	Toilet floor	66	**0 ± 0**	**11 ± 0.4**	**0 ± 0**	15 ± 1.1	26 ± 0.0	**0 ± 0**	16 ± 3.2	**0 ± 0**	16 ± 4.3	27 ± 0	20 ± 0.0	**0 ± 0**	27 ± 2.0
16	AKTH_180	Drawer	180	**0 ± 0**	27 ± 2.2	**0 ± 0**	15 ± 0.0	29 ± 1.3	**0 ± 0**	15 ± 2.2	**0 ± 0**	14 ± 2.2	23 ± 2.5	30 ± 2.2	**0 ± 0**	**0 ± 0**
17	AKT_259	Urine	180	**0 ± 0**	18 ± 1.1	18 ± 2.2	14 ± 0.0	22 ± 3.4	15 ± 1	20 ± 1.1	**0 ± 0**	18 ± 0.0	28 ± 0.5	0 ± 0	21 ± 1.3	23 ± 1.2
18	AKTH_311	Bed swab	180	**0 ± 0**	19 ± 2.2	12 ± 3.4	17 ± 0.4	10 ± 1.1	**0 ± 0**	16 ± 1.2	**0 ± 0**	21 ± 1.3	**0 ± 0**	22 ± 0.0	**0 ± 0**	**0 ± 0**
19	BB_1	Soil	180	**11 ± 1.6**	20 ± 1.3	20 ± 1.5	14 ± 1.1	26 ± 1.0	17 ± 1.2	18 ± 2.2	**0 ± 0**	15 ± 2.8	12 ± 1.5	28 ± 1.5	22 ± 1.4	32 ± 1.4
20	DH_21	Door handle	180	**11 ± 0.6**	14 ± 0.0	15 ± 0.8	**13 ± 3.0**	25 ± 2.2	16 ± 0.8	16 ± 0.8	**0 ± 0**	17 ± 0.2	21 ± 0.3	22 ± 0.5	23 ± 0.0	28 ± 2.2
21	DH_24	Door handle	180	13 ± 0.4	16 ± 0.0	0 ± 0	16 ± 2.2	23 ± 4.2	**0 ± 0**	20 ± 1.2	**0 ± 0**	20 ± 1.4	24 ± 0	19 ± 2.2	19 ± 0.0	28 ± 0.5
22	DH_34	Door handle	180	**0 ± 0**	18 ± 0.2	0 ± 0	**13 ± 1.8**	25 ± 2.4	**0 ± 0**	16 ± 2.0	**0 ± 0**	13 ± 3.3	22 ± 0	24 ± 2.2	21 ± 1.5	8 ± 0.0
23	TF_31	Toilet floor	180	14 ± 2.2	16 ± 2.8	0 ± 0	15 ± 0.6	22 ± 0.0	**0 ± 0**	18 ± 1.4	**0 ± 0**	22 ± 0.9	22 ± 0	23 ± 0.5	14 ± 0.5	12 ± 1.6
24	TF_50	Toilet floor	180	**0 ± 0**	**11 ± 0.8**	20 ± 2.2	15 ± 0.4	26 ± 1.0	18 ± 1.2	16 ± 2.2	**0 ± 0**	24 ± 2.4	27 ± 0	21 ± 1.5	23 ± 0.5	28 ± 0.4
25	TF_53	Toilet floor	180	**8 ± 1.2**	**11 ± 0.2**	0 ± 0	**11 ± 0**	20 ± 0.8	**0 ± 0**	20 ± 1.0	**0 ± 0**	18 ± 3.1	29 ± 1.6	18 ± 0.0	18 ± 1.6	26 ± 2.2
26	TF_54	Toilet floor	180	14 ± 0.1	18 ± 1.4	0 ± 0	19 ± 1.0	18 ± 3.5	**0 ± 0**	21 ± 2.1	**0 ± 0**	22 ± 4.2	32 ± 0.4	15 ± 0.0	23 ± 1.6	28 ± 0.0
27	TF_9	Toilet floor	180	**10 ± 0**	**11 ± 2.3**	18 ± 0	13 ± 0.4	24 ± 1.9	16 ± 1.4	18 ± 1.1	**0 ± 0**	21 ± 3.2	28 ± 0.7	16 ± 2.4	**0 ± 0**	26 ± 1.4

^*C*chloramphenicol, *CN*gentamicin, *K*kanamycin, *CT*colistin, *IMP*imipenem, *TE*tetracycline, *TGE*tigecycline, *AMP*ampicillin, *RD*rifampicin, *CAZ*ceftazidime, *CIP*ciprofloxacin, *PTB*piperacillin−tazobactam, *FEP*cefepime^

### Acquired Antibiotic Resistance Genes in *A. baumannii* Strains

Using ResFinder and BacWGSTdb databases, acquired antibiotic resistance were detected in-silico in 22 *A. baumannii* isolates using the WGS data. In addition, the virulence gene predictions were performed using the BacWGSTdb. The selection of the 22 was randomly done to represent variety of sources they were isolated from. A total of 8 different resistance genes were identified (Table 4), most of which are genes encoding aminoglycoside, tetracycline, sulphonamide, and beta-lactam resistance. The Ambler class C β-lactamases, *Acinetobacter*-derived cephalosporinase*bla*_ADC-25_ gene was found in all *A. baumannii* strains (*n* = 22; 100%), while the class D carbapenemase*bla*_OXA-66_ was found in 16 (59.2%), and *bla*_OXA-180_ in 11 (40.7%). As for the aminoglycoside resistance, 5 distinct aminoglycoside encoding resistance genes (*aad*A1, *aph*(3′′)-Ib, *aac*(3)-Ia, *aph*(6)-Id, *aph*(3′)-Ia) were detected and are present in all strains harbouring *bla*_OXA-66_ (Supplementary Table S2). Sulphonamide resistance was predominantly encoded by *sul*1 and *sul*2 (*n* = 16; 4.07%). Similarly, 16 isolates (8 from hospital environment, 5 from toilet floor, 1 from door handle, and 2 from sullage) harboured *tetB* genes, but 6 are susceptible to tetracycline phenotypically.

**Table 4:** Intrinsic and acquired antibiotic resistance genes in *A. baumannii* isolated from hospital and non-hospital environments

Sample iD	Aminoglycoside	Beta-lactam	Tetracycline	Sulphonamide
AKTH_180 AKTH_212 DH_20 MMSH_13 MMSH_43 MMSH_50 MMSH_69 MMSH_73 MMSH_80 SULL_24 SULL_60 TF_10 TF_13 TF_27 TF_30 TF_45	*aad*A1, *aph*(3′′)-Ib, *aac*(3)-Ia, *aph*(6)-Id, *aph*(3′)-Ia	*bla*_ADC-25_, *bla*_OXA-66_	*tetB*	*sul1*, *sul2*
AKTH_311 AKTH_259 BB_1 DH_21 DH_24 DH_34 TF_9 TF_31 TF_50 TF_53 TF_54		*bla*_OXA-180_, *bla*_ADC-25_		

*aph*(3′)−Ia=neomycin, kanamycin, lividomycin, paromomycin, ribostamycin, *aph*(3′′)−Ib=streptomycin, *aph*(6)−Id=streptomycin, *aac*(3)−Ia=gentamicin, astromicin, fortimicin, *aadA*1=spectinomycin, streptomycin, *tetB*=doxycycline, tetracycline, minocycline

39 virulence genes were detected in all *bla*_OXA-66_ variants, but *bla*_OXA-180_ variants are deficient in at least 2 virulence genes. A *bap* virulence gene, which plays a role in biofilm formation, is present in only 15 isolates and absent in others (7). Quorum-sensing gene *abaR* that coordinates the expression of specific genes as a function of population density, and a gene encoding a membrane fusion protein, *adeF* were absent only in MMSH_13. The virulence gene *basI,* which regulates heme utilization, was absent in MMSH_50. Similarly, *csuA*, which plays a central role in initial bacterial attachment and biofilm formation on abiotic surfaces, is absent in MMSH_50, MMSH_80, TF_45, and *csuB* is absent in DH_20 (Supplementary Table S1).

In addition, *bauA*, which is a virulence gene involved in the synthesis and transport of small, iron chelating molecules (siderophores), is present in all except BB_1, TF_50, TF_54, DH_24 (environmental samples), and AKTH_259 (a hospital sample).

### MLST Analyses to Determine Local Epidemiology of *A. baumannii*

MLST analyses based on the Oxford scheme identified 2 sequence types (ST), with each found in both hospital and non-hospital environments. Sixteen out of the 22 isolates belong to ST^Oxf^ 1050/ 2058 (the duplication of *gdhB* locus was the reason for having 2 ST^Oxf^ 1050 and 2058 [31]), while the remaining 7 had MLST allelic profiles of ST^Oxf^942. In addition, 8 out of the 16 isolates with ST^Oxf^ 1050/2058 are isolates recovered from hospital environment, while others were isolates recovered from sullage (Sull_24, 60) and toilet floors of student hostels (TF_10, 13, 27, 30, 45) which are several kilometers apart from the hospitals. On the other hand, isolates with ST^Oxf^942 were all from environmental sources (non-hospital source) except isolate AKTH_259, which is from the hospital environment. The genomes of the 22 isolates were submitted to the PubMLST database with submission ID BIGSdb_20220824235613_030441_37368. Similarly, MLST analyses based on the Pasteur scheme also identified 2 sequence types (ST^Pas^ 2 and ST^Pas^ 267). The 16 strains with ST^Oxf^ 1050/2058 belong to ST^Pas^ 2, and those with ST^Oxf^ 942 have ST^Pas^267 (Table 5).

**Table 5:** Sequence type (ST) of 22 *A. baumannii* according to Oxford and Pasteur schemes

Gene bank Biosample/accession N^o^	Sample ID	Strain name	Sample source	*bla*OXA51 variant	MLST SCHEME	
					Oxford	Pasteur
SAMN30755304	TF_45	IY-BUK-21	Surface (non-hospital)	66	1050, 2058	2
SAMN30755303	MMSH_69	IY-BUK-20	Hospital environment	66	1050, 2058	2
SAMN30755302	MMSH_50	IY-BUK-19	Hospital environment	66	1050, 2058	2
SAMN30755301	TF_30	IY-BUK-18	Surface (non-hospital)	66	1050, 2058	2
SAMN30755300	SULL_24	IY-BUK-17	Sullage	66	1050, 2058	2
SAMN30755299	SULL_60	IY-BUK-16	Sullage	66	1050, 2058	2
SAMN30755298	DH_24	IY-BUK-15	Surface (non-hospital)	180	942	267
SAMN30755297	TF_53	IY-BUK-14	Surface (non-hospital)	180	942	267
SAMN30755296	TF_50	IY-BUK-13	Surface (non-hospital)	180	942	267
SAMN30755295	DH_21	IY-BUK-12	Surface (non-hospital)	180	942	267
SAMN30755294	TF_9	IY-BUK-11	Surface (non-hospital)	180	942	267
SAMN30755293	MMSH_73	IY-BUK-10	Hospital environment	66	1050, 2058	2
SAMN30755292	MMSH_80	IY-BUK-9	Hospital environment	66	1050, 2058	2
SAMN30755291	TF_13	IY-BUK-8	Surface (non-hospital)	66	1050, 2058	2
SAMN30755290	DH_20	IY-BUK-7	Door handle (non- hospital)	66	1050, 2058	2
SAMN30755289	TF_10	IY-BUK-6	Surface (non-hospital)	66	1050, 2058	2
SAMN30755288	AK_259	IY-BUK-5	Clinical (hospital)	180	942	267
SAMN30755287	AK_212	IY-BUK-4	Surface (hospital)	66	1050, 2058	2
SAMN30755286	BB_1	IY-BUK-3	Soil (non-hospital)	180	942	267
SAMN30755285	AK_180	IY-BUK-2	Surface (hospital)	180	1050, 2058	2
SAMN30755284	MMSH_43	IY-BUK-1b	Hospital environment	66	1050, 2058	2
SAMN30755283	MMSH_13	IY-BUK-1a	Hospital environment	66	1050, 2058	2

**Fig. 2:**
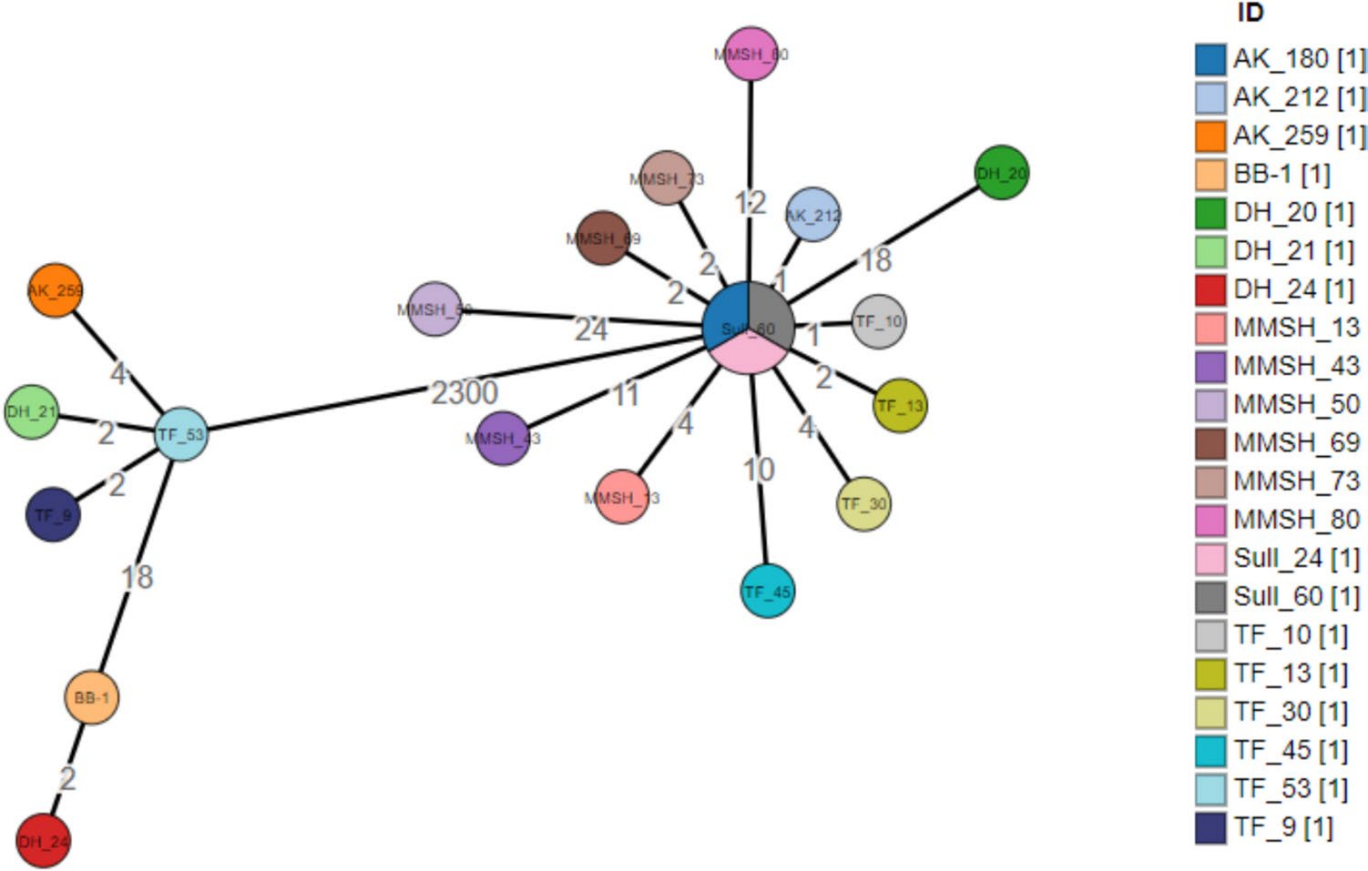
Minimum spanning tree based on cgMLST illustrates two clusters, represented by ST 2 and 267 according to the Pasteur scheme. The figure was generated using the bacWGSTdb online tool. At connecting lines, the number of differing alleles between the isolates connected is indicated (logarithmic scale)

A minimum spanning tree based on cgMLST clustered *bla*_OXA-66_ variants into one clade and *bla*_OXA-180_ variants into another (Fig. 2). The two clusters are separated by 2300 alleles. Within each cluster, the allelic distances range from 2 to 18 for the *bla*_OXA-180_ cluster and 1–24 for the *bla*_OXA-66_ cluster. BB_1, isolated from a semi-pristine environment (ecological garden), is notably distant from all other isolates in the *bla*_OXA-180_ cluster, with an allelic distance of 18.

The core genome phylogeny constructed was found to comprise approximately 9047 genes shared among all analyzed isolates. Of these, core and soft core genes were 2498 and 362, respectively, while shell and cloud genes were, respectively, 1385 and 4802 (Supplementary Fig. S1).

### Mobile Genetic Elements (MGEs) Analysis

Plasmid detection with PlasmidFinder did not yield positive result. However, a further search with MobSuites revealed mixture of predicted mobilizable and none mobilizable plasmids with varying size. Plasmid mediated AMR genes (*n* = 7, of different genes) which confer resistance to aminoglycosides, tetracycline, sulphonamides, gentamicin, sisomicin, fortimicin, kanamycin, neomycin, streptomycin were detected. A mobilizable plasmid of about 137 Kb which carried 4 ARGs was detected in samples isolated from MMSH_50 and MMSH_80. Majority of the plasmids from both hospital and non-hospital isolates harbor a transferable *sul2* gene (Table 6). Some of the *bla*_OXA-66_ variants harbor IS*Aba*1 upstream of *bla*_OXA51 like_ genes, which indicate their phenotypic resistance. In addition, functional IS*Aba*2, IS*Aba*18, IS*Aba*33, IS*Aba*125 are widely spread throughout the genome (Table 7).

**Table 6:** List of predicted plasmids, ARGs, and their predicted transferability identified in *A. baumannii* strains and their closest relatives found in GenBank

Sample ID	Size (bp)	AMR genes	predicted transferability	Total No. of predicted plasmids	No. mobilizable	Plasmid name based on NCBI	% identity	coverage
MMSH_80	137,501	Aph(3′′)-Ib, Aph(6)-Id, Sul2, tet(B)	Mobilizable	4	1	pV_JAB108, pCFSAN093708	100	100
TF_45	115,866	Sul2	Mobilizable	4	1	pWM99c-2	100	100
MMSH_69	118,925	Sul2	Mobilizable	3	1	pWM99c-2, pAYP-A2	99.98	100
TF_13	118,887	Sul2	Mobilizable	3	1	pV_JAB108,	99.99	100
DH_20	121,686	Sul2	Mobilizable	5	1	pAYP-A2	100	100
AKTH_259			Non mobilizable	4	0	NA	NA	NA
TF_30	118,661	Sul2	Mobilizable	4	1	pCFSAN093707	100	100
MMSH_43	121,953	Sul2	mobilizable	4	1	pV_JAB108,	100	100
MMSH_50	136,791	Sul2, blaADC30	Mobilizable	7	1	pV_JAB108,	100	100
TF_10	119,961	Sul2	Mobilizable	2	1	pCFSAN093707	100	100
AKTH_180	119,957	Sul2	Mobilizable	2	1	pWM99c-2	100	100
TF_53	NA	NA	Non mobilizable	3	0	NA	NA	NA
DH_24	99,765	NA	Non mobilizable	3	0	NA	NA	NA
TF_50		NA	Non mobilizable	3	0	NA	NA	NA
TF_13	119,931	Sul2	Mobilizable	4	1	pV_JAB108	99.99	100
MMSH_73	118,732	Sul2	Mobilizable	2	1	pCFSAN093707	100	100
AKTH_212	118,757	Sul 2	Mobilizable	3	1	pCFSAN093707	100	100
DH_21	99,740	NA	Non mobilizable	3	0	NA	NA	NA
	46,720							
TF_9	47,600	NA	Non mobilizable	4	0	NA	NA	NA
MMSH_13	118,707	Sul 2	Mobilizable	4	1	pCFSAN093707	100	100
BB_1	99,761	NA	Non mobilizable	3	0	NA	NA	NA
	48,691							
Sull_24	119,998	Sul2	Mobilizable	2	1	pCFSAN093707	100	100
Sull_60	118,811	Sul2	Mobilizable	2	1	pCFSAN093707	100	100
TF_27	118,675	Sul 2	Mobilizable	3	1	pV_JAB108	100	100

^*NA*not available^

**Table 7:** Insertion sequences identified in *A. baumannii* isolates using IS finder

Sample ID	*Bla*_OXA51_ variants	IS*Aba* type								
		1	2	12	13	18	33	59	62	125
AKTH_212	66	+	+			+	+			+
DH_20	66	+	+	+	+	+	+	+	+	+
MMSH_13	66	+	+		+	+	+	+	+	+
MMSH_ 43	66		+	+		+	+		+	+
MMSH_50	66	+	+		+	+	+	+	+	+
MMSH_69	66	+	+		+	+	+	+	+	+
MMSH_73	66	+	+		+	+	+	+	+	+
MMSH_80	66	+	+		+	+	+	+	+	+
Sull_24	66	+	+		+	+	+	+	+	+
Sull_60	66	+	+		+	+	+	+	+	+
TF_10	66	+	+		+	+	+	+	+	+
TF_13	66	+	+		+	+	+	+	+	+
TF_27	66	+	+		+	+	+	+	+	+
TF_30	66	+	+		+	+	+	+	+	+
TF_45	66	+	+		+	+	+	+	+	+
AKTH_180	180	+	+	+	+	+	+	+	+	+
AKTH_259	180	+	+	+	+	+	+	+	+	+
BB_1	180	+	+	+	+	+	+	+	+	+
DH_21	180	+	+	+	+	+	+	+	+	+
DH_24	180	+	+	+	+	+	+	+	+	+
TF_50	180	+	+	+	+	+	+	+	+	+
TF_53	180	+	+	+	+	+	+	+	+	+
TF_9	180	+	+	+	+	+	+	+	+	+

## Discussion

To understand the genomic epidemiology of *A. baumannii* strains in Kano, Nigeria, we studied using WGS, the local strains of *A. baumannii* circulating in hospital and non-hospital environments, and clinical samples in Kano state of Nigeria.

In this study, a 19.8% prevalence of *Acinetobacter* spp and 15.7%* A. baumannii* was detected in samples collected over 6 months. This aligns with reports by Anane et al., who observed a 19% prevalence of *Acinetobacter* species in extra-hospital environments in South Africa [32], and Deborah et al. who recovered *A. baumannii* from two-thirds of rooms housing patients in Singapore [33]. Differences in recovery rates between hospital and non-hospital sources may reflect variations in human activity exposure, especially in congested areas.

Molecular typing of *bla*_*OXA-51*_-like genes revealed two variants: *bla*_OXA-66_ (associated with international clone 2, IC2) and the rare *bla*_OXA-180_. These were recovered across diverse sources, including hospital (DH, TF, and Sull) and non-hospital environments (bed surfaces, chairs, and drawers), suggesting they are the main circulating variants. The detection of *bla*_OXA-66_ in hospital samples supports its established link to globally disseminated IC2 clones in healthcare settings. However, its presence in environmental samples, where it is less frequently reported, raises questions about its transmission. Hospital waste and wastewater systems, rich in bacteria and antimicrobial residues, could serve as vectors introducing *bla*_OXA-66_ into the environment.

This finding suggests a potential interaction between clinical and environmental reservoirs, warranting further investigation into its survival outside hospitals. The isolation of IC2 clones from multiple non-hospital sources supports the hypothesis that *A. baumannii* is prevalent in the environment rather than simply escaping frowm hospitals. Recent studies also highlight IC2 clones transitioning from hospitals to natural environments, such as water systems [34–36].

Conversely, *bla*_*OXA-180*_, which is rarely reported globally, was detected in environmental samples and, notably, in hospital settings, including patient urine sample. This finding is significant, as *bla*_*OXA-180*_ isolates generally have limited resistance profiles, with susceptibility to nearly all antibiotics except those mediated by their intrinsic resistance genes including *bla*_*ADC-25*_and *bla*_*OXA-180*_. The presence of *bla*_*OXA-180*_ variants in hospital settings, especially in clinical samples, suggests that these strains may have entered healthcare environments through environmental reservoirs or human activity. The association of *bla*_*OXA-180*_ variants with hospital environments, despite their minimal resistance profiles, raises questions about their fitness and potential to establish a foothold in healthcare settings. ST^Pas^267 was first isolated as community-acquired bacteria causing bacteraemic infection in an Indigenous Australian [37], but now more frequently reported in developing countries such as Ethiopia [38], Tanzania [38] and Haiti [38] between 2010 and 2016, until recently when it was isolated in Germany from milk powder [39]. The sequence types (STs) identified in this study differ from those previously reported in hospital wastewater in Southwest Nigeria, as described by Odih et al. [40]. This variation suggests possible geographical differences in the distribution of *A. baumannii* clones and could indicate the potential influence of local antimicrobial usage practices, healthcare infrastructure, or other environmental factors on the clonal expansion of resistant strains. The absence of overlap in dominant STs further emphasizes the need for geographically broad surveillance to fully understand the dynamics of antimicrobial resistance across different regions in Nigeria.

**Fig. 3:**
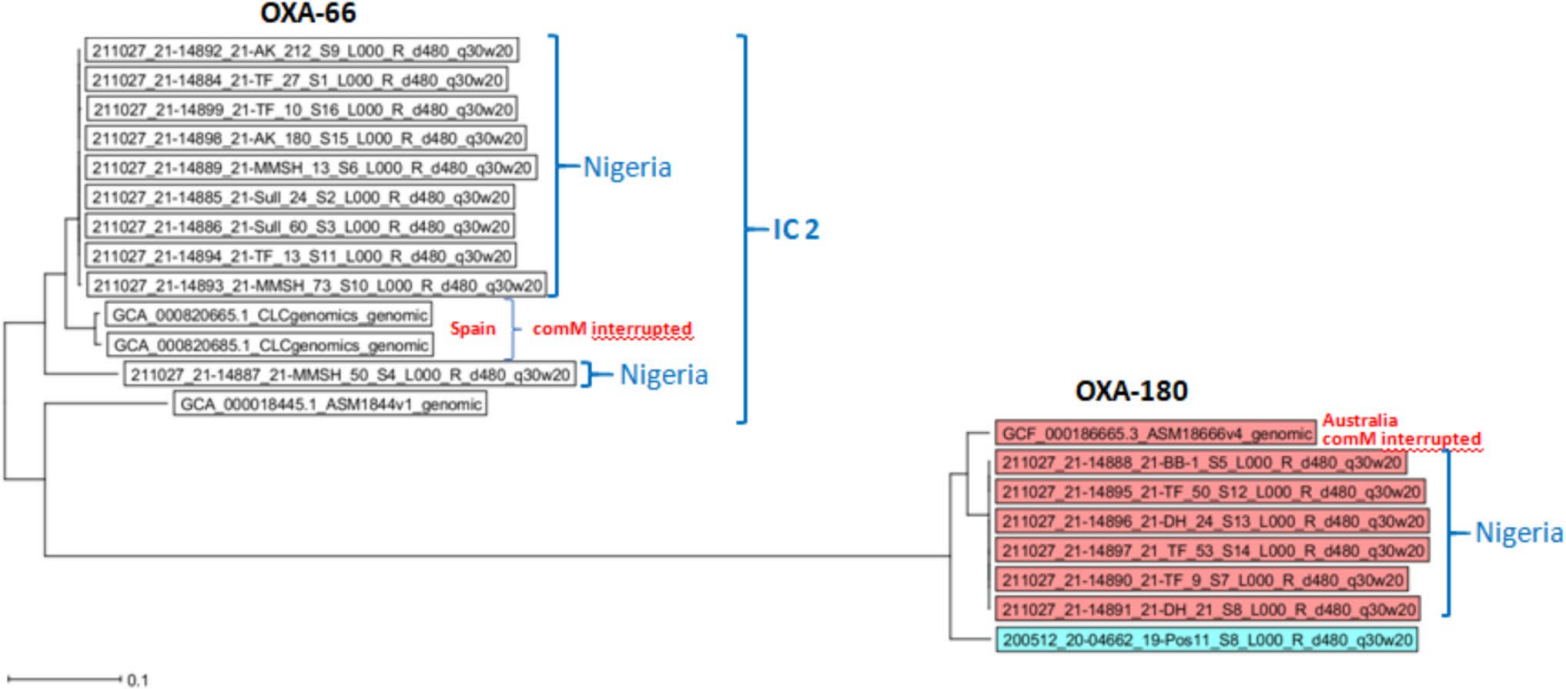
Phylogenetic clustering of *Acinetobacter baumannii* isolates based on *blaOXA* variants. Isolates with *blaOXA-66* variants from Nigeria cluster with a Spanish variant featuring intact *commM*, while isolates with *blaOXA-180* variants cluster with an Australian isolate exhibiting interrupted *commM*

Phylogenetic analysis reveals that all the *bla*_*OXA-180*_ (ST^Pas^267) variant form clusters with two intriguing international strains: a Polish strain isolated from a white stork (a migratory bird species) (ST^Pas^267) and a clinical Australian strain. The Polish isolate’s origin from a migratory bird suggests the possibility of long-range dispersal of *A. baumannii* through natural ecological pathways, such as bird migration and airborne. The Australian strain, with an interrupted *comM* genomic island (Fig. 3), represents a different evolutionary context, suggesting that these isolates may be adapting independently to local conditions. Interestingly, all ST^Pas^267 (*bla*_OXA-180_ variant) isolates in this study contained intact *comM.* genes. However, clinical isolates, including those from IC2, typically accumulate resistance genes on a specific island integrated into the *comM* locus [41]. The absence of this resistance island in our IC2 representatives (ST^Pas^2, *bla*_OXA-66_) suggests they have not been present in the hospital environment for an extended period.

The core genome phylogeny tree revealed the presence of at least three major clades, which reflects genetic diversity among the isolates. Clade I contained primarily isolates from environmental sources (typical bla_*OXA-180*_ variants/ST2), suggesting a possible environmental lineage. Clade II included isolates closely related to ST2 strains known possibly indicating recent divergence from environmental strains and/or contamination or environmental persistence of clinical lineages. Clade III was dominated by isolates from semi-pristine soils, potentially representing less exposed or native strains. The clustering pattern was supported by high bootstrap values across internal branches, confirming the phylogenetic relationships among the strains.

Another notable observation was high resistance of ST^Pas^2/*bla*_OXA-66_ strains to chloramphenicol and piperacillin/tazobactam. Further, *sul*1and *sul*2 genes that mediate resistance to sulfonamides were present in all the *bla*_OXA-66_ variants, but absent in strains with ST^Pas^267.

All ST^Pas^2 (*bla*_OXA-66_) from hospital and non-hospital environments carry genes encoding resistance to β-lactams, aminoglycosides, sulfonamides, and tetracyclines. However, fewer acquired genes were observed compared to literature reports, where genes for fosfomycin, fluoroquinolones, monobactams, and phenicols are common in hospital *A. baumannii* strains. Notably, ST^Pas^267 (*bla*_OXA-180_ variants) did not acquire additional resistance genes, even in hospital strains, aligning with reports of community soil-isolated *A. baumannii* being largely antibiotic-susceptible [42]. Although the *tetB* gene was found in all ST^Pas^2/*bla*_OXA-66_ isolates, 2 DH isolates were susceptible to tetracycline. Recovery of ST^Pas^267 (*bla*_OXA-180_) from an inpatient highlights potential environment-to-human transmission. Plasmids play a key role in spreading pathogenicity and resistance genes in bacteria. No plasmids were detected in any isolates (even with a minimum 20% coverage) using the PlasmidFinder database, likely due to its lack of specific scheme for identifying plasmids in *Acinetobacter spp.* However, with MobSuite mobilizable plasmids were found in both hospital and non-hospital isolates, indicating potential HGT across environments. ST2 isolates showed higher ARG diversity, reflecting strong selective pressures in healthcare settings in comparison with ST267. In addition, ST267 isolates were mainly associated with *sul2*, conferring sulfonamide resistance.

The study shows that *A. baumannii* in Kano (Northwest) hospital environments differs from strains in Southwest Nigeria, which are more diverse and highly resistant. However, detailed source information on two isolates collected from Ibadan, Nigeria, listed in the PubMLST database as ST^Pas^267 and ST^Oxf^2473, was not available for comparison [13]. The reason for this diversity is not known presently, but differences in ecological factors, including weather, could play a significant role [43].

The fact that ecological garden and hostels locations are close and that SNP distances between isolate from BB_1 and those from DH, TF, and Sull range from 80 to 750 indicates the establishment of the strains in the environment for longer periods and that strains recovered from DH of hostel room was recently introduced into the door handle environment. These observations, particularly in the light of the time distance between isolation sources, indicate existence of transmissibility of the 2 variants between the locations. The origin of these clones is not known, but it seems likely that they dwell in the environment (sullage) and then diverge into other environments within short periods of time.

## Conclusion

The study revealed a 15.7% prevalence of *A. baumannii* in both hospital and non-hospital environments in Kano, Northwest Nigeria. The local strains identified in Kano appear distinct from those reported in other regions of Nigeria. Two major sequence types (ST^Pas^2 and ST^Pas^267harbouring*bla*_OXA-66_ and *bla*_OXA-180_, respectively, are widely distributed in both clinical and environmental settings. Interestingly, ST^Pas^267/*bla*_OXA-180_which was primarily found in environmental samples, was also detected in urine sample of a patient, which raises concerns about the role of environmental reservoirs in the spread of this lineage in Kano, Nigeria. The persistent of ST^Pas^267/*bla*_OXA-180_in hospital environment settings suggests they could adapt to healthcare environments. ST^Pas^2/*bla*_OXA-66_, associated with globally disseminated IC2 clone, clustered with other strains from other region, which indicates their adaptability to different environmental niches. ST^Pas^267/*bla*_OXA-180_clustered with Polish and Australian strains, which indicate long-range dissemination through natural ecological pathways, such as migratory birds, or other mechanisms. The results in this study suggest the interconnectedness of clinical and environmental reservoirs in the propagation of *A. baumannii*. This suggests the need for continuous genomic surveillance and integrated public health interventions to mitigate the spread of these variants and their associated resistance genes across environments.

## Supplementary Information

Below is the link to the electronic supplementary material.

Supplementary file1 (DOCX 674 KB)

Supplementary file2 (XLSX 36 KB)

Supplementary file3 (XLSX 24 KB)

## Data Availability

Data supporting the findings of this study are freely available as supplementary tables and figures, and whole genome sequences are available in GenBank. The whole genome sequences of the isolates were deposited in NCBI under the BioProject accession number PRJNA878801, with BioSample numbers SAMN30755283 to SAMN30755304.
