# Supplementary figures and images for: Local Genomic Epidemiology of Acinetobacter baumannii Circulating in Hospital and Non-hospital Environments in Kano, Northwest Nigeria
Source: Curr Microbiol. 2025 Jun 8;82(7):329. doi: 10.1007/s00284-025-04304-z (PMC12146226; doi:10.1007/s00284-025-04304-z)

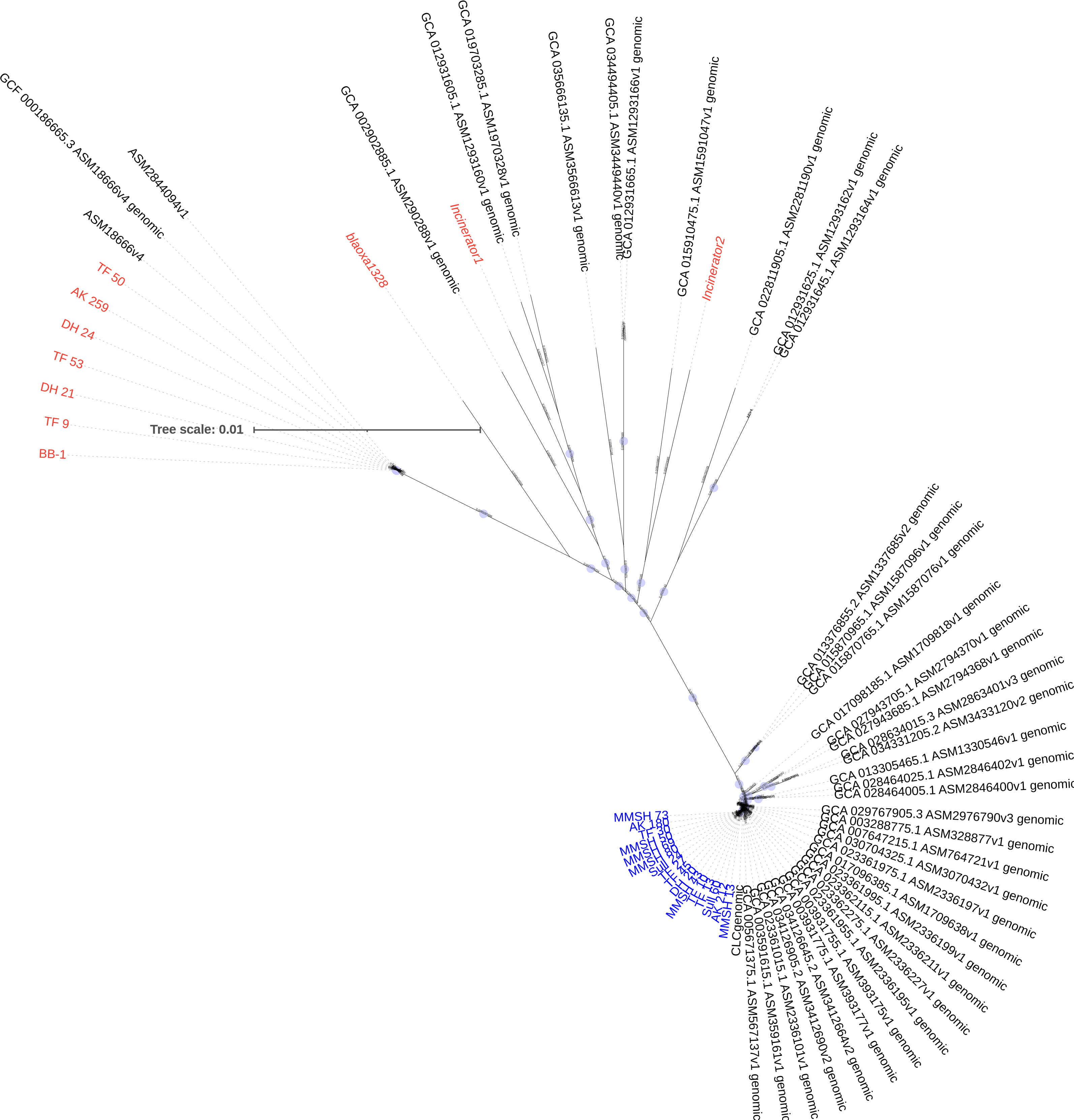

Supplement: Supplementary file 1 — Supplementary file1 (DOCX 674 KB) [file 284_2025_4304_MOESM1_ESM.docx]
